# A Data Driven Approach to Assess Complex Colour Profiles in Plant Tissues

**DOI:** 10.3389/fpls.2021.808138

**Published:** 2022-01-26

**Authors:** Peter Andrew McAtee, Simona Nardozza, Annette Richardson, Mark Wohlers, Robert James Schaffer

**Affiliations:** ^1^The New Zealand Institute for Plant & Food Research (PFR), Auckland, New Zealand; ^2^The New Zealand Institute for Plant & Food Research (PFR), Kerikeri, New Zealand; ^3^The New Zealand Institute for Plant & Food Research (PFR), Motueka, New Zealand; ^4^School of Biological Sciences, University of Auckland, Auckland, New Zealand

**Keywords:** computer vision, fruit, plant, colour analysis, quantification of colour, region-growing algorithm, growing

## Abstract

The ability to quantify the colour of fruit is extremely important for a number of applied fields including plant breeding, postharvest assessment, and consumer quality assessment. Fruit and other plant organs display highly complex colour patterning. This complexity makes it challenging to compare and contrast colours in an accurate and time efficient manner. Multiple methodologies exist that attempt to digitally quantify colour in complex images but these either require *a priori* knowledge to assign colours to a particular bin, or fit the colours present within segment of the colour space into a single colour value using a thresholding approach. A major drawback of these methodologies is that, through the process of averaging, they tend to synthetically generate values that may not exist within the context of the original image. As such, to date there are no published methodologies that assess colour patterning using a data driven approach. In this study we present a methodology to acquire and process digital images of biological samples that contain complex colour gradients. The CIE (Commission Internationale de l’Eclairage/International Commission on Illumination) ΔE2000 formula was used to determine the perceptually unique colours (PUC) within images of fruit containing complex colour gradients. This process, on average, resulted in a 98% reduction in colour values from the number of unique colours (UC) in the original image. This data driven procedure summarised the colour data values while maintaining a linear relationship with the normalised colour complexity contained in the total image. A weighted ΔE2000 distance metric was used to generate a distance matrix and facilitated clustering of summarised colour data. Clustering showed that our data driven methodology has the ability to group these complex images into their respective binomial families while maintaining the ability to detect subtle colour differences. This methodology was also able to differentiate closely related images. We provide a high quality set of complex biological images that span the visual spectrum that can be used in future colorimetric research to benchmark colourimetric method development.

## Introduction

Phenotyping is an important scientific field that involves quantifying the observable physical properties of an organism. In plant science there is a constant upward pressure to produce increasingly accurate and precise phenotyping strategies to empower a high-resolution understanding of the genetic, metabolic, and transcriptomic drivers of plant phenotypes (Furbank and Tester, 2011; Cobb et al., 2013; Dhondt et al., 2013; Mutka and Bart, 2014; Rahaman et al., 2015; Yamamoto et al., 2017; Ilahy et al., 2019; Pieruschka and Schurr, 2019). Due to its economic, health, and cultural significance, colour in plants has been studied for over 100 years (Kraemer, 1906; Farago et al., 2018). Plant organs show a huge diversity of colours, with some organs such as flowers and fruit often displaying complex colour patterns. Different colours often extend across the tissue, cellular, and subcellular levels of the plant organ (Noda et al., 1994; Lister et al., 1996; Rat’kin, 2000; Tan et al., 2013). Plants control the expression of colour through the accumulation of pigments (biochromes) that are often located within the organelles of the cells (Mol et al., 1998; Moreau et al., 2012). They can also control colour by manipulating the pH of the intracellular space containing the pigment and through the organisation of pigment containing cell layers (Lancaster, 1992; Noda et al., 1994; Liu et al., 2015). Fruit often display a wide range of colour patterning in their external tissues, with differences observed between the exposed blush and shaded non-blush sides of fruit (Lancaster, 1992; Delgado-Pelayo et al., 2014) as well as between the external and internal tissues of an organ (Espley et al., 2007). Colour patterning can also vary between different species of the same genus and even between different cultivars of the same species (Goodrich et al., 1992; De Jong et al., 2004; Moreau et al., 2012; Zhao et al., 2012; Ilahy et al., 2015; Iwashina and Mizuno, 2020).

In a biological context, colour can be used by plants as a signalling mechanism to other organisms that might interact with the plant (Chittka and Menzel, 1992; Koes et al., 1994; Clegg and Durbin, 2000). These interactions can include the inhibition of predation of leaves, the attraction of pollinators to flowers, or in the case of fruit, as a cue to potential seed dispersers that the fruit is ready to be consumed. In addition to displaying colouration differences between organs, some plants are also capable of changing the colour of an organ during development and/or in response to an environmental cue (Lancaster, 1992; Shen et al., 2011; Liu et al., 2015). The diverse range of colour polymorphism observed in the plant kingdom is particularly evident in flowers and fruit but is also present in the stems, leaves, tubers, and roots.

It is still common for academic and industrial studies to grade the colour of plant organs using subjective visual scales involving human participants. This is a challenging task to achieve in a precise or objective manner due to the complex nature of colour patterns observed in plant organs like fruit (Thierry et al., 2008). There are also significant analytical challenges when comparing human observed colour data due to variation between individual participants in both the type and the number of photoreceptors used to detect colour (Nathans et al., 1986). Other factors that can influence the perception of colour by humans include recall memory, retinal fatigue, and interactive effects of the background and shape of the object (Perez-Carpinell et al., 1998; Bloj et al., 1999; Duffy and Chan, 2002). These factors make it difficult to objectively compare human perceived colour data with simple patterning. The complex and multifaceted nature of plant colour composition makes accurate description of human perceived colour data largely impossible.

Multiple research methodologies have been employed to circumvent the human perception of colour (Delgado-Pelayo et al., 2014). Some studies have aimed to directly measure the chemical composition of plant biochromes within the cells of sampled tissue. These studies attempted to use the quantified chemical compound(s) as a proxy to estimate the colour. This methodology assumes linearity between the quantity of the chemical and physiological presentation of the colour and does not account for the 2-dimensional or 3-dimensional distribution of biochromes within the plant organ. Advances in digital technologies have offered an alternative approach to quantify and compare colour attributes. Some commonly used tools include the Konica Minolta Chroma-meter that digitally captures the reflectance of light within a known radial area. These tools output a device-independent trinary CIE 1976 (L*a*b*) coordinate that describes the colour of the object by averaging the reflectance values within the measured area (Richardson et al., 2011; Khairi et al., 2015; Logvinenko, 2015). This type of colour measuring system is suitable when assessing an object with a high degree of uniformity in colour but is less useful when trying to assess the complex colour patterning observed in fruit.

Most commercially available image capture devices output data in the device-dependent RGB colour space. The RGB colour space contains more than 16 million colours making any comparison on a colour-by-colour basis between samples prohibitively long in terms of computational time (Lazier, 1953; Zeileis et al., 2009; Logvinenko, 2015). An additional challenge for these types of sensors is the incident light source itself can affect the perception of colour (reflected light). Fortunately this can be compensated for by including an internal colour standard within the context of the acquired image and by controlling the ambient light source using a controlled environment (imaging-box) (Kendal et al., 2013).

Currently, multiple methodologies exist that attempt to digitally quantify colour in images but these either require *a priori* knowledge to assign colours to a particular bin (Huang et al., 2010; Ngo and Macabebe, 2016), or average the colours present within a assayed region into a single colour value (Fullerton and Keiding, 1997). In this study we developed a standardised method to measure colour and a data driven methodology to summarise and quantify different colour patterning in cross sectional fruit/tuber images, greatly simplifying the complexity of colours identified in the images. The colour data simplification using this novel methodology were used to rebuild virtual images as a validation process.

## Materials and Data Collection

### Biological Samples

A diverse selection of 28 species of fruit and tubers was purchased from a local supermarket in Auckland, New Zealand. These fruit and tubers represented different families including Anacardiaceae (mango), Ebenaceae (persimmon), Actinidiaceae (kiwifruit), Lauraceae (avocado), Musaceae (banana), Rosaceae (apple, peach, pear, plum, and strawberry), Rutaceae (grapefruit, lemon, mandarin, and orange), and Solanaceae (potato, tamarillo, and tomato). Each fruit was cross sectioned along its most symmetrical side. Up to three cross sections of the same fruit type were placed face down on the scanner on a predefined 3 × 1 grid with defined positions to allow image capture of the individual fruit.

In a more detailed experiment to evaluate the effect of light exclusion on anthocyanin production in fruit flesh, 100 *Actinidia chinensis* var. *chinensis* ‘Zes008’ (red-fleshed kiwifruit) were also studied. These fruit were harvested from a commercial orchard based in Kerikeri New Zealand (for details see Richardson et al., 2021). During fruit growth these fruit were either bagged with lightproof bags (Hirst et al., 1990) from 56 days after anthesis (DAA) for 108 days or left unbagged (control).

### Image Capture

A Canon LIDE 220 flatbed scanner (Scanning element sensor: CIS, Light source: 3 colour RGB LED) was placed in a 2 mm black perspex box with a retractable lid to completely block ambient light. A transparent A4 sized sheet with a printed 3 × 1 grid (140 mm × 95 mm) was placed on the scanner platen along with an internal colour standard in the form of an X-Rite mini-colour checker card (Mccamy et al., 1976). An image with dimensions of 4,960 pixels (W) and 7,015 pixels (H) was acquired at a resolution of 600 dots-per-inch/pixels-per-inch (DPI/PPI) and output in a TIFF format. Copies of each TIFF format image were then resaved in PNG and JPEG formats for comparison.

### Colour Standardisation and Segmentation

The flatbed scanner used in this study used the device dependent RGB colour space to denote colour value. It is well-established practice that images are standardised to a control even in controlled lighting conditions for differences in sensor sensitivity. Fiji/ImageJ (version 1.52p) (Alam et al., 2014) was used to calibrate the colour images using the white tile standard on the X-Rite mini-colour checker card that was included in each scanned image. Each fruit was then segmented from its parent image using a colour thresholding method that excluded the background colour (black) and saved as an independent sibling image in TIFF format. Publicly available copies of these images can be found at https://www.kaggle.com/petermcatee/colorimetry-standard-fruit-images.

## Method and Algorithm Description

### Colorimetric Analysis

Analysis of colour data values was done on sibling images segmented from the background using the open source software R (v3.6.1) (R-Core-Team, 2020). Two colour space transcoding processes were used in this study. A transcode between the gamut of RGB colour values in each image to the hexadecimal syntax was done using the *rgb* function supplied by the *grDevices* (v3.6.2) package (R-Core-Team, 2020). A transcode between the gamut of RGB colour values into the CIE 1976 (L*a*b*) space was achieved using the *convertColor* function tools supplied as part of the *grDevices* (v3.6.2) package. This transformation used the standard illuminant at D65 with XYZ tristimulus values normalised for relative luminance [0.9504, 1.0000, 1.0888]. K-means clustering performed in this study used the *kmeans* function provided in the *stats* (v3.6.2) package. The initialisation method used for k-means clustering was “Hartigan-Wong.”

The ΔE2000 distance measure was calculated using the CIE 1976 (L*a*b*) transcoded colour data and the *deltaE2000* function supplied as part of the *colorscience* (v1.0.8) package (Gama and Davis, 2019). This equation was used to group colour values based on similarity using the region-growing algorithm, quantify differences/similarities between *pixel-for-pixel* comparisons of colour values between file formats, and to cluster images using the weighted CIE ΔE2000 distance metric.

**FIGURE 1:**
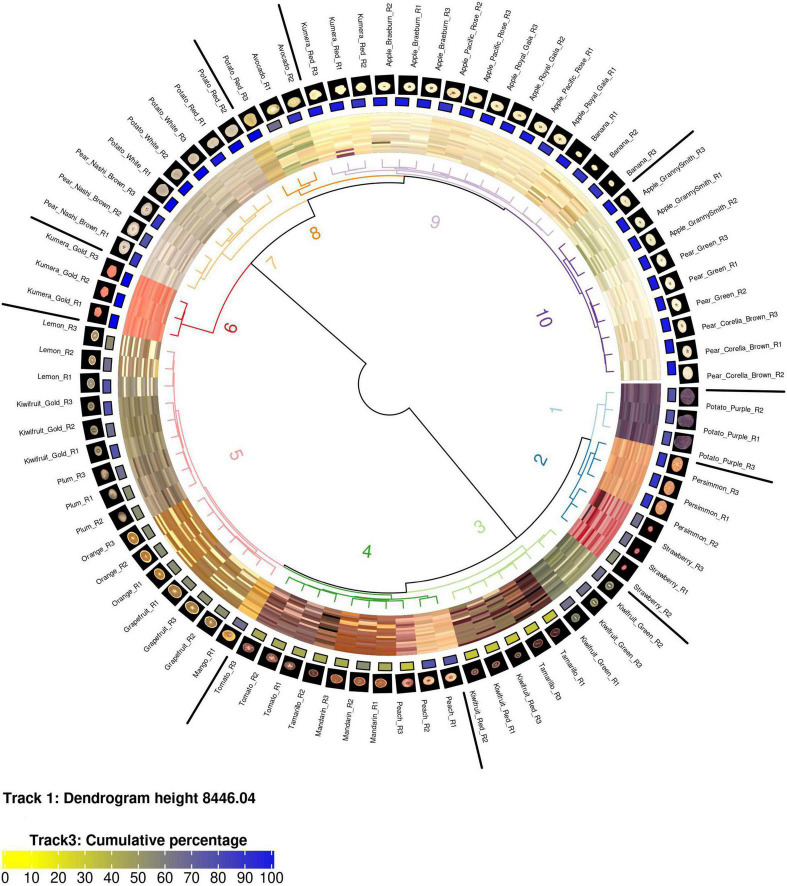
A circularised diagram displaying the clustering of fruit using the weighted ΔE2000 distance metric. From the centre, track 1 shows a dendrogram of the clustered region grown data using the weighted ΔE2000 distance metric against all colour values per fruit. Track 2 displays a visualisation matrix consisting of the top 20 colours summarised by the region-growing algorithm for each respective fruit. Track 3 shows the cumulative percentage of the top 20 colours for each respective fruit. Track 4 displays the raw image for each fruit. Track 5 indicates the name of each fruit. R, biological replicate.

**FIGURE 2:**
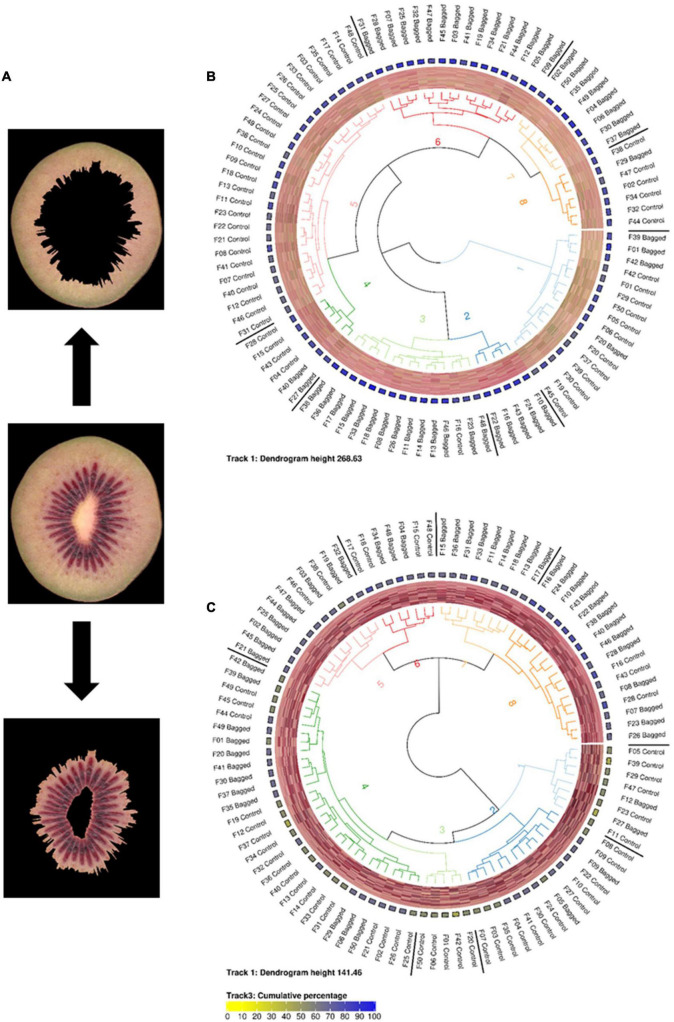
**(A)** Representative images of segmented inner and outer pericarp tissues from *Actinidia chinensis* var. *chinensis* ‘Zes008’ kiwifruit. Fruit in this study were either treated with bagging or not (Control) to evaluate the effect of light exclusion on anthocyanin production (red colour accumulation) in fruit flesh. Circularised diagrams display the clustering of data from outer **(B)** and inner **(C)** pericarp segments using the weighted ΔE2000 distance metric. From the centre, track 1 shows a dendrogram of the clustered region-grown data using the weighted ΔE2000 distance metric against all colour values per fruit. Track 2 displays a visualisation matrix consisting of the top 20 colours summarised by the region-growing algorithm for each respective fruit. Track 3 shows the cumulative percentage of the top 20 colours for each respective fruit. Track 4 indicates the name of each fruit.

Tools supplied as part of the *EBImage* package were used to parse, modify, and save image files (Pau et al., 2010). The *circlize* package was used to generate the circular dendrograms presented in Figures 1, 2 (Gu et al., 2014).

### Region Growing Algorithm

The region-growing algorithm was developed and implemented in R version 3.6. This algorithm begins by prioritising the most frequent unique colour (UC) values within an image. The most frequent UC value is set as a seed and it is recursively tested against all the other UC values (queries) using the CIE ΔE2000 formula. If the result of this calculation is below a threshold value (for example ≤ 2) then the frequency of the query value is reassigned to the seed value and the query value is removed from the pool. This process repeats until all the UC values have been tested and/or reassigned. The final output is a list of perceptually unique colours (PUC) and their frequencies (as a percentage of the total image) known as a PUC-table. The pseudocode for the region-growing algorithm is presented in Supplementary Data Sheet 1.

### Recolouring of Images for Validation Purposes

Recolouring of images was also done using R version 3.6 and the *EBImage* package. This process uses the region-growing algorithm stated above. It tests the colour values of all the pixels in an image and reassigns the colour value of a pixel if the CIE ΔE2000 distance (relative to a tested seed colour) is below the defined threshold value. In this manner all the colours in an image are reassigned the value of their perceptually unique bin and a perceptually unique raster of the image is made. The pseudocode for recolouring images is outlined in Supplementary Data Sheet 1.

### Weighted CIE ΔE2000 Distance Metric

The weighted CIE ΔE2000 distance metric was developed and implemented in R version 3.6. This metric works by using the “perceptually unique colour (PUC) tables” generated by the region-growing algorithm. A “transport matrix” is constructed by undertaking a full-rank comparison of all of the PUC values contained between two PUC-tables (images). The CIE ΔE2000 distance function is used to calculate a distance metric between each respective colour comparison. A ‘weighed distance value’ is then generated by multiplying the ΔE2000 distance value by the difference in frequency (as a percentage) of the respective PUC comparison. The closest match to a PUC value is considered as the minimum weighed distance value contained in each row of the “transport matrix.” The overall similarity between two PUC profiles (all the values in a PUC-table) can then be considered as the sum of the minimum weighed distance values (closest matches). This is similar to Wasserstein metric but uses the CIE ΔE2000 formula to define distance and factors in the frequency/abundance difference between colour values. The pseudocode that was used to generate the CIE ΔE2000 weighted distance matrix can be found in Supplementary Data Sheet 1.

## Results

### Scanner Based Imaging

To standardise the colour measurements, a flatbed scanner was used within a purpose built light-proof box. This setup was developed as it provided a consistent light source that was unaffected by changes in the ambient light. The box was of sufficient depth to allow space for a thick layer of plant tissue to be placed on the scanner without admitting any light through the sample. Using this setup, the cross sections of 28 diverse fruit and tubers were scanned and stored in TIFF file format for analysis.

### Assessment of Image Formats

**FIGURE 3:**
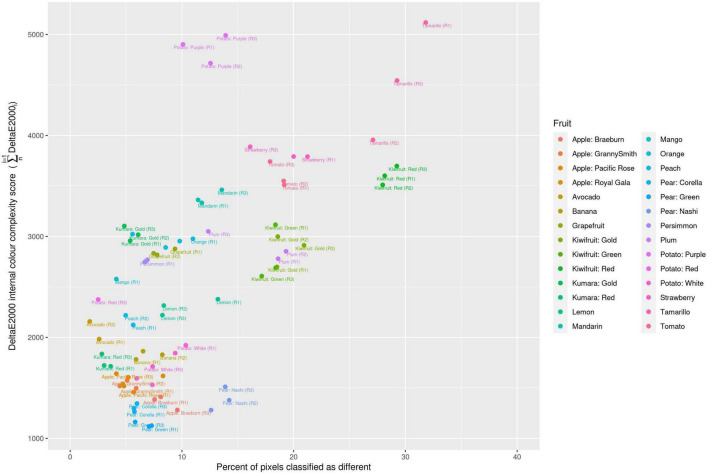
Classification of different colour values due to JPEG format compression compared with the colour complexity score of the fruit. R, biological replicate.

**FIGURE 4:**
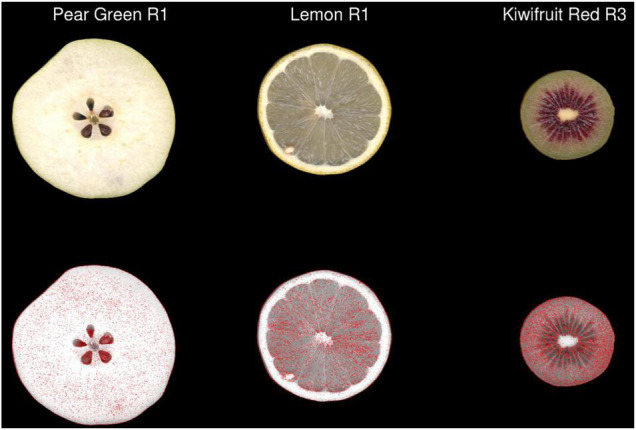
The 2-dimensional distribution of pixels classified as having different colour values in JPEG format compared with TIFF format in three representative images of fruit. Pixels containing a ΔE2000 distance >2 between each format are coloured red in the second row. R, biological replicate.

Two different image file formats were compared: lossless (TIFF) and lossy (JPEG). A *pixel-for-pixel* comparison showed that JPEG compression had a significant effect on the colour values. This compression effect varied between different images. The lowest and highest mean mismatch percentages were observed in avocado and tamarillo images with mean mismatch percentages of 2.17 and 29.40%, respectively (Table 1). The effect of the JPEG compression algorithm on mismatch frequency increased with increasing colour complexity of the fruit, however, this relationship was not linear (Figure 3). To identify the location at which colour differences occurred in these image formats, three representative images where selected according to their low mismatch (Pear Green R1), mid-range mismatch (Lemon R1), and high mismatch (Kiwifruit Red R3) frequency. The two dimensional distribution of mismatches in JPEG images tended to occur in regions of the fruit where the colour gradient changed (Figure 4). In all images, there was a higher amount of mismatches at the intersection of the background and foreground. No predictable pattern of mismatches was observed within each subject image.

**TABLE 1:** A comparison between the JPEG and PNG file formats.

Subject type	Identical in JPEG	Similar in JPEG	Different in JPEG	Identical in PNG
Apple ‘Braeburn’	2.42 ± 0.05	89.17 ± 0.05	8.42 ± 0.61	100
Apple ‘Granny Smith’	1.42 ± 0.08	93.44 ± 0.08	5.14 ± 0.43	100
Apple ‘Pacific Rose’	2.11 ± 0.16	92.88 ± 0.16	5.01 ± 0.46	100
Apple ‘Royal Gala’	2.36 ± 0.30	91.70 ± 0.3	5.94 ± 1.18	100
Avocado	3.50 ± 0.09	94.33 ± 0.09	2.17 ± 0.42	100
Banana	2.68 ± 0.19	90.43 ± 0.19	6.89 ± 0.70	100
Grapefruit	1.68 ± 0.14	90.11 ± 0.14	8.21 ± 0.59	100
Kiwifruit Gold	1.05 ± 0.02	79.61 ± 0.02	19.34 ± 0.79	100
Kiwifruit Green	1.18 ± 0.07	80.86 ± 0.07	17.97 ± 0.41	100
Kiwifruit Red	0.57 ± 0.02	70.98 ± 0.02	28.45 ± 0.39	100
Kumara Gold	2.15 ± 0.05	92.42 ± 0.05	5.43 ± 0.36	100
Kumara Red	5.98 ± 1.09	90.86 ± 1.09	3.17 ± 0.24	100
Lemon	2.31 ± 0.26	87.74 ± 0.26	9.95 ± 1.63	100
Mandarin	0.97 ± 0.05	86.77 ± 0.05	12.26 ± 0.66	100
Mango	1.71 ± *NA*	94.16 ± *NA*	4.13 ± *NA*	100
Orange	1.44 ± 0.11	88.79 ± 0.11	9.78 ± 0.71	100
Peach	1.92 ± 0.06	92.69 ± 0.06	5.40 ± 0.22	100
Pear ‘Corella’ Brown	2.72 ± 0.21	91.48 ± 0.21	5.81 ± 0.08	100
Pear Green	3.39 ± 0.03	89.89 ± 0.03	6.72 ± 0.46	100
Pear Nashi Brown	1.53 ± 0.04	84.9 ± 0.04	13.57 ± 0.49	100
Persimmon	1.03 ± 0.04	92.17 ± 0.04	6.8 ± 0.07	100
Plum	1.43 ± 0.18	81.81 ± 0.18	16.76 ± 2.21	100
Potato Purple	1.21 ± 0.12	86.6 ± 0.12	12.19 ± 1.12	100
Potato Red	2.25 ± 0.33	92.48 ± 0.33	5.27 ± 1.44	100
Potato White	1.88 ± 0.1	89.08 ± 0.1	9.04 ± 0.88	100
Strawberry	0.57 ± 0.05	80.32 ± 0.05	19.11 ± 1.55	100
Tamarillo	0.67 ± 0.04	69.94 ± 0.04	29.39 ± 1.36	100
Tomato	0.86 ± 0.03	80.42 ± 0.03	18.72 ± 0.41	100

*This table summarises the number of pixels that were identical, similar, or different in each comparison. Data are presented as mean ± standard error and represent three biological replicates for each class of fruit/tuber with the exception of mango for which only one fruit was sampled.*

### Colorimetric Analysis of Fruit/Tuber Images

**FIGURE 5:**
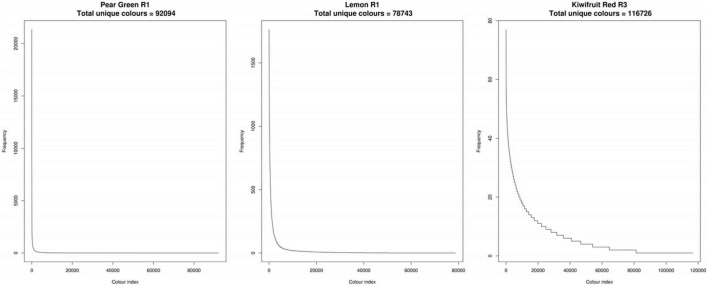
Ordered frequency distributions of unique colour values for three representative fruit. R, biological replicate.

To assess colour variability in the images, the colour values of each pixel from the TIFF format images were transcoded to hexadecimal code. Frequency tables of the hexadecimal codes were then constructed to determine the number of unique colour (UC) values in each subject image (Supplementary Table 1). The number of UC values within each subject imaged was highly variable among the fruit analysed in this study. The lowest number of UC values was observed in Potato White R3, with 34,629 values and the highest number of UC values was observed in Mandarin R2, with 202,377 values. Ordered frequency distributions demonstrated that the majority of hexadecimal codes had a frequency of <10 values across all of the subject images used in this study (Figure 5). This demonstrated that there are a large number of subtle colour differences within the plant tissues.

To simplify the colour space data, a region-growing algorithm was developed to condense the colour descriptors within each subject image into perceptually unique colours (PUC) in an unbiased manner. The algorithm reduced the density of colorimetric data points on average by 98%. The highest number of colour values after the region-growing algorithm was applied, using a threshold ΔE2000 value of 2, was 3,078 (Supplementary Table 1). Applying an additional threshold to the summarised region-grown colour values that contributed >0.1% of the total percentage of each image reduced the data density on average by 99.9% with a maximum of 219 colour values. The biggest determinant of the processing time was the number of UC values that each raw image contained (Supplementary Table 2).

To determine the impact on the overall processing time and compression of the data, the ΔE2000 threshold of the region-growing algorithm was tested at 1, 1.5, 2, 2.5, and 3 (Supplementary Table 2). Relative to a ΔE2000 threshold of 2, increasing the threshold to 2.5 and 3 reduced processing time on average by a ratio of 0.86 and 0.71, respectively. In terms of the number of output colour bins, increasing the ΔE2000 threshold to 2.5 and 3 reduced the average number of colour bins by a ratio 0.59 and 0.38, respectively, relative to a threshold of 2. Relative to a ΔE2000 threshold of 2, decreasing the ΔE2000 threshold to 1.5 and 1 increased processing time 1.5 and 2.9 fold, respectively. This also increased the number of output colour bins by a ratio of 2 and 4.6, respectively.

### Comparison With K-Means Clustering

The region-growing methodology was compared with the widely used k-means clustering methodology in order to assess its processing efficiency. The region-growing algorithm (at ΔE2000 threshold = 2) was faster than k-means clustering using a K-value of 20 or 100, with ratios in processing time of 0.95 and 0.79, respectively (Supplementary Table 2).

**FIGURE 6:**
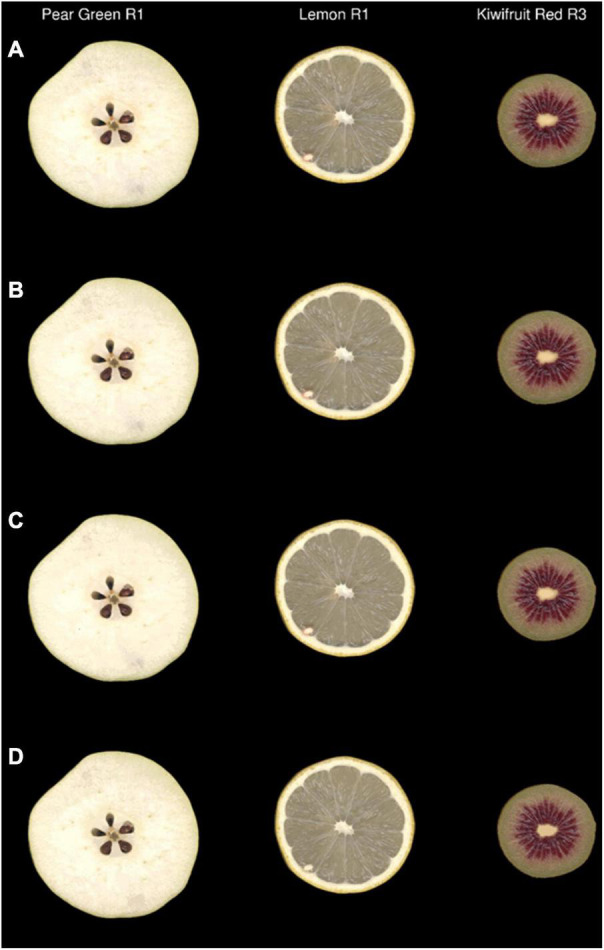
Original images **(A)** and simulated images of three representative fruit that were recoloured using the output colour bins generated by the region-growing algorithm with ΔE2000 at thresholds of 2 **(B)** and 3 **(C)** or by k-means clustering (*K* = 20) **(D)** R, biological replicate.

To back validate the efficiency of the colour data clustering generated from the region-growing and k-means cluster analysis, the outputs of these algorithms were used to raster new images as described in the Method and Algorithm Description section. An example of this process can be seen in Figure 6, which shows that there was a high degree of visual similarity between the original image and the images generated using output colour bins generated at ΔE2000 thresholds of 2 and 3. Inspection of the k-means-generated images showed considerable loss of colour texture within each image (Figure 6D and Supplementary Figure 1). This was particularly evident in the seeds of the apple, around the skin of the lemon, and at the central core of the kiwifruit.

Image compression value was used to determine how well the original image complexity was represented by the region grown and k-means clustered algorithms. The region-growing algorithm resulted in linear regression values of 0.90, 0.84, 0.80, 0.76, and 0.72 for ΔE2000 thresholds of 1, 1.5, 2, 2.5, and 3, respectively. The k-means clustering algorithm resulted in linear regression values of 0.71 and 0.71 at K values of 20 and 100, respectively (Supplementary Table 2).

### Clustering of Region-Grown Data Across a Broad Spectrum of Colours

The weighted ΔE2000 distance metric was used to generate a distance matrix using the region-grown colour output for each image. Hierarchical clustering of the weighted distance matrix was used to cluster the data into 10 groups (Figure 1). A dendrogram, with the respective images aligned, shows that each of the 10 clusters was associated with a different colour attribute. For example, the “lighter” orange hue associated within Cluster 5 grouped orange, mango, and grapefruit together and this was different from the “darker” orange hue that separated mandarin into Cluster 4. In general, all fruit types were co-localised with the exception of Tamarillo R2 and Potato Red R3. Visual inspection of the Tamarillo R2 image showed that is contained a greater amount of “lighter” orange hues compared to the other tamarillo replicate images. Visual inspection of the Potato Red R3 showed that it contained a greater amount of the yellow/orange colour hues compared with its replicate images. In both these cases the outlier images were visually distinct from their respective replicates, which demonstrates the need for replicates when undertaking colour studies.

### Fruit With Different Colour Patterns

To extend the utility of this methodology into fruit with similar but complex patterns, a larger experiment using 100 red fleshed kiwifruit *Actinidia chinensis* var. *chinesis* (‘Zes008’) was undertaken. Red fleshed kiwifruit often have an intense inner pericarp red colour and a lighter outer pericarp colour (Wang et al., 2003). In other species such as apple, exclusion of environmental light is known to increase red pigment (anthocyanin) accumulation in the skin, though less is known about the effect of light exclusion on internal fruit tissues. To test if exclusion of environmental light affects the internal fruit tissues of kiwifruit, a total of 50 fruit were bagged to induce red colour accumulation while 50 control fruit were left unbagged.

In order to differentiate the colour values located in the outer and inner pericarp of kiwifruit images, a script was developed to automatically identify the boundaries of these tissue zones and segment the inner and outer pericarp tissue regions of fruit. This allowed the lighter coloured outer pericarp to be separated from the darker red of the inner pericarp (Figure 2A).

These segmented images were then summarised using the region-growing algorithm and clustered using the weighted ΔE2000 metric. To assess variability within the condensed colour space (PUC) profiles the inner and outer pericarp tissues were K-means clustered. The outer pericarp showed greater variability in colour profiles (maximum height of the dendrogram was 268.63) compared with the inner pericarp (maximum height of 141.46) (Figures 2A,B). Surprisingly, the outer pericarp clusters 2, 3, and 6 (associated with bagged fruit) contained greater amounts of red in their colour profiles. The outer pericarp colour profiles of unbagged fruit were largely localised in clusters 1, 5, and 8 and were associated with more yellow/green colour profiles.

## Discussion

The analysis of colour patterning of complex images like fruit can be a time consuming and subjective process. Our approach aimed to develop a data driven and time efficient methodology that could summarise the diverse colour patterning observed in nature by clustering colour values that have no perceptible difference from each other. In this study we selected fruit types to encompass a wide variety of colours across the visible spectrum. The amount of unique colours within the images used in this study ranged from tens of thousands to hundreds of thousands. The relatively high density of colour data points within each image made it challenging to summarise the data to an amount that was unbiased and still meaningful to human interpretation. The CIE ΔE2000 formula provided a reliable basis to measure the perceptual distance between colours in the L*a*b* colour space (Sharma et al., 2005; Gijsenij et al., 2011). The formula was used in this study to measure generalised colour complexity and formed the basis for developing the region-growing algorithm. ΔE2000 values below 2 are regarded as having minimal perceptual difference by the human eye while values >2 but <3 are regarded as being distinguishable by the human eye at a glance (Witzel et al., 1973; Mokrzycki and Tatol, 2011). These are, however, generalised rules and it should be noted that there is a wide range of variation in the perceptive ability of individuals (Nathans et al., 1986).

K-means clustering or segmentation of the colour gamut into predefined bins are two of the most common approaches to speed up the processing time of digital colorimetric analysis of images (Witzel et al., 1973; Celebi, 2011; Bharathi and Subashini, 2013; Wang et al., 2019). These methodologies output arrays in regular formats that are easy to compare, but have the drawback of requiring *a priori* knowledge of a predefined number of bins to which to assign each colour. Additionally, both of these approaches describe the colour value for a given bin as an average value that might not exist within the context of the original dataset. The region-growing approach developed in this study solves this issue by using a data driven approach to independently interrogate and grow the optimal number of colour bins for each image. This data driven approach is unbiased but has the drawback of having an irregular output array. The weighted ΔE2000 distance metric is a robust method that allowed us to measure the distance between the irregular output arrays (colour profiles) produced by the region-growing algorithm. The concept of this metric was based on the Earth Mover’s Distance (EMD) metric which is commonly used to measure differences between colour profiles (Rubner et al., 2000; Yu and Herman, 2005; Takada and Yanai, 2008; Weller and Westneat, 2019). EMD uses a Euclidean distance metric which poorly approximates the colour space a human perceives. The EMD-like process (weighted ΔE2000 distance metric) developed in this publication instead uses the ΔE2000 formula to calculate distance. Our analysis demonstrates the utility of this mathematical method to cluster images based on their region-grown outputs. We were able to cluster images while maintaining the ability to detect subtle colour differences that are commonly observed in biological samples such as fruit. This can be most notably observed in the dissimilar cluster localisation of Potato Red R2 and Tamarillo R2 images outside of the clusters containing their respective replicates and is further demonstrated by the comparison of fruit with close visual similarity shown in Figure 2. We observed a stronger regression score for images generated using the region-grown methodology than for those generated via k-means clustering, when compared with the compression rate of the original image. This indicates that colour value outputs of the region-growing methodology better approximated those observed in the original image than did outputs generated by the k-means analysis. Additionally, visual inspection of the region-grown *in silico* simulated images showed more consistency in their visual similarity to the original than those generated by k-means clustering. This is perhaps unsurprising as the k-means clustering approach is often subjected to under or over fitting on data, particularly when the overall complexity of the data is not known in advance, as is the case in biological images.

Interestingly, there is relatively little published research that attempts to quantify the effect that image compression algorithms have on the colour values within an image. The two types of data compression algorithms that are used by image acquisition tools are lossless (TIFF and PNG) and lossy (JPEG). Lossless image compression has the ability to restore the data to its original value after decompression but produces relatively large image files with higher storage overheads (Murray and Vanryper, 1996). Lossy compression does not allow full restoration of the original data values but produces much smaller image files, thereby reducing storage overheads (Murray and Vanryper, 1996). Selecting the most suitable image format is the first decision step in an image analysis pipeline and ultimately can influence the downstream analysis of the image. Here we compared three image formats (TIFF, PNG, and JPEG) to quantify the effect of different data compression algorithms on colour values from complex biological images, and showed that uncompressed files are the only way to maintain the complexity of data in fruit images. While the small file sizes produced by JPEG compression are attractive from a perspective of file storage, this compression affects the integrity of the data and is likely to have implications for applications that require precision colour estimation including machine learning applications.

## Conclusion

This publication demonstrates the utility of a data driven approach for the analysis of images with complex colour profiles. We first developed a novel algorithm to reduce the density of data within each image in a process called region growing. The processing time of this algorithm was comparable with other commonly used methodologies such as K-means clustering. One major advantage of our approach is that it does not require *a priori* knowledge of the amount of K-mean bins to generate. This is important for the comparison of images based on their colour features as it avoids subjective under/over fitting of data. Central to our methodology was the use of the previously published CIE ΔE2000 formula. This formula was used to quantify the difference between colours in a colour-space that aligns with human perceptive ability. This is different from other methodologies that use the Euclidean formula to measure differences in colour data.

We also developed a novel process to compare the region-grown colour profiles from a dataset with diverse colour patterning and from a separate dataset with visually similar colour patterning. The utility of this methodology could have significant implications towards improving the accuracy of computer vision phenotyping of plant tissues particularly in regard to colourimetric analysis. Applications of this methodology could extend across various plant related academic and research disciplines including plant breeding, and postharvest quality assessment.

## Data Availability Statement

The datasets presented in this study can be found in online repositories. The names of the repository/repositories and accession number(s) can be found in the article/Supplementary Material.

## Author Contributions

RS and PM conceived the project and wrote the manuscript. RS and SN obtained the funding. PM, AR, and SN generated the data. PM developed the algorithm, which were checked and improved by MW. All authors checked and edited the final manuscript.

## Conflict of Interest

The authors declare that the research was conducted in the absence of any commercial or financial relationships that could be construed as a potential conflict of interest.

## Publisher’s Note

All claims expressed in this article are solely those of the authors and do not necessarily represent those of their affiliated organizations, or those of the publisher, the editors and the reviewers. Any product that may be evaluated in this article, or claim that may be made by its manufacturer, is not guaranteed or endorsed by the publisher.

## Supplementary Material

Supplementary Figure 1 Side-by-side comparison of avocado images recoloured with data processed with region-growing or k-means methodologies. The textural differences in colour are most pronounced between the k-means and the original image.

## Abbreviations

ΔE2000: A mathematical formula used for measuring the similarity of tristimulus values. The purpose of creating the ΔE2000 metric was to measure colour differences more accurately and in a manner that is perceptually uniform with human observation
CIE: Commission Internationale de l’Eclairage/International Commission on Illumination
PUC: Perceptually unique colours
PUC-table: A table containing the hexadecimal codes of the perceptually unique colours generated using the region-growing algorithm. Also included in this table is the corresponding percentage cover of the subject that assigned in each PUC
RGB: Tristimulus colour components (Red, Green, Blue)
UC: Unique colours.
