# Supplementary figures and images for: A Data Driven Approach to Assess Complex Colour Profiles in Plant Tissues
Source: Front Plant Sci. 2022 Jan 26;12:808138. doi: 10.3389/fpls.2021.808138 (PMC8826216; doi:10.3389/fpls.2021.808138)

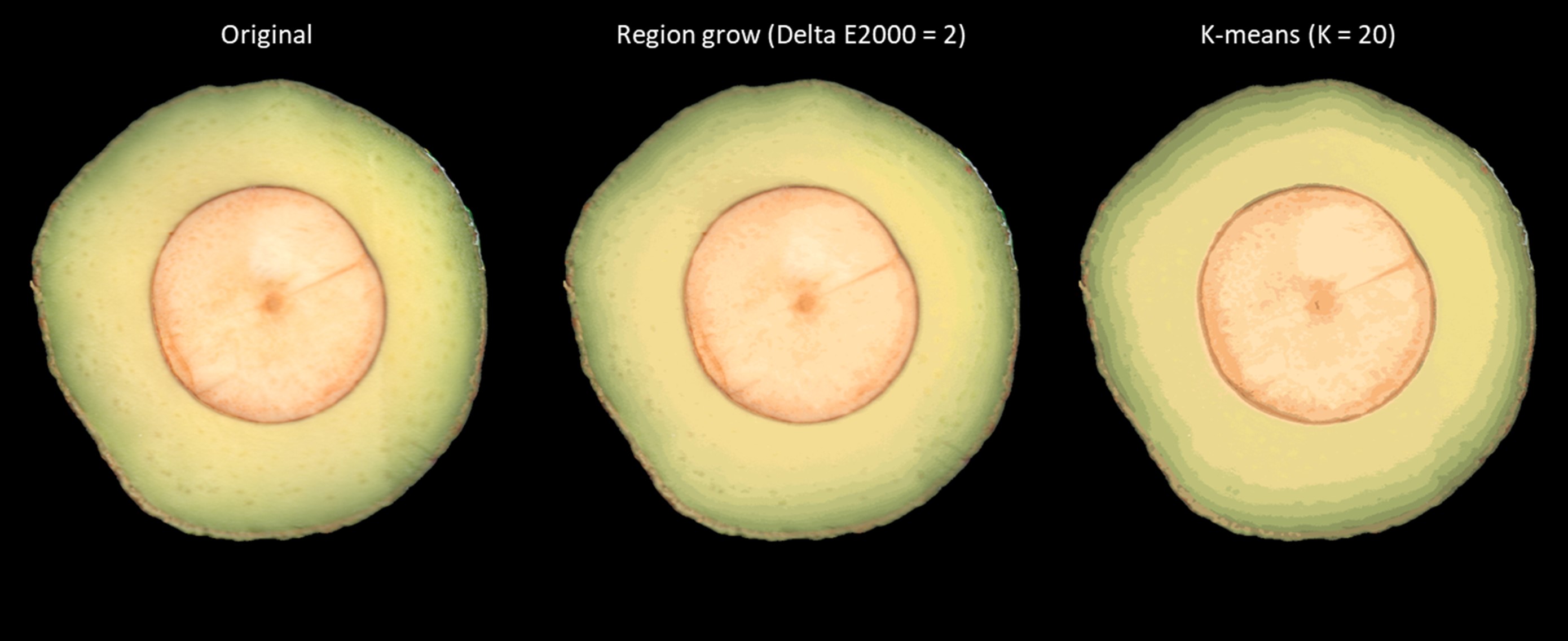

Supplement: Supplementary Figure 1 — Side-by-side comparison of avocado images recoloured with data processed with region-growing or k-means methodologies. The textural differences in colour are most pronounced between the k-means and the original image. [file Image_1.JPEG]
