# Supplementary material for: A Data Driven Approach to Assess Complex Colour Profiles in Plant Tissues
Source: Front Plant Sci. 2022 Jan 26;12:808138. doi: 10.3389/fpls.2021.808138 (PMC8826216; doi:10.3389/fpls.2021.808138)
Supplement: Supplementary file 4 [file Data_Sheet_1.docx]

## 3.2 Region growing algorithm

The region-growing algorithm was developed and implemented in R version 3.6. This algorithm begins by prioritising the most frequent unique colour (UC) values within an image. The most frequent UC value is set as a seed and it is recursively tested against all the other UC values (queries) using the CIE ΔE2000 formula. If the result of this calculation is below a threshold value (for example <=2) then the frequency of the query value is reassigned to the seed value and the query value is removed from the pool. This process repeats until all the UC values have been tested and/or reassigned. The final output is a list of perceptually unique colours (PUC) and their frequencies (as a percentage of the total image) known as a PUC-table. The pseudocode for the process is outlined below.

Declare double: *d, F_sNorm_*

Declare vector (character): *vec_hex*

Declare vector (double): *vec_freq*

Declare *L*a*b** vector (double): *L_q_*, *L_t_*

Declare character: *H_s_, H_q_*

Declare integer *n, F_t_*, *F_s_*, *F_q_*

1. Convert the RGB pixel values to a vector of hexadecimal values
2. Generate a frequency table of unique hexadecimal codes (*vec_hex and vec_freq*)
3. Rank *vec_hex and vec_freq* in descending order of frequency

*vec_hex* and *vec_freq* now contain corresponding ordered hexadecimal codes and frequencies

1. Set *f_t_* = sum of all frequencies
2. Set *n_s_* = number of hexadecimal codes
3. Set *n_q_* = number of hexadecimal codes
4. Loop: Repeat for less than or equal to *n_s_*

Loop: Repeat for less than or equal to *n_q_*

Set *d* = 0

Set *f* = 0

Set *H_s_* = *vec_hex* [*n_s_*] (seed hexadecimal code)

Set *H_q_* = *vec_hex* [*n_q_*] (query hexadecimal code)

Set *F_s_* = *vec_freq* [*n_s_*] (seed frequency)

Set *F_q_* = *vec_freq* [*n_q_*] (query frequency)

Covert *H_s_ and H_q_ to* L*a*b* colour space (*L_s_* and *L_q_)*

Calculate *d* using ΔE2000 equation between *L_s_* and *L_q_*

if *d* <= 2 then

Calculate *F_s_* = *F_s_* + *F_q_*

Remove *vec_hex = vec_hex* [-*n_q_*]

Remove *vec_freq = vec_freq* [-*n_q_*]

End loop

Calculate *F_sNorm_* = *F_s_/ F_t_*100*

Write to output *H_s_* and *F_sNorm_* as *Region Grow data*

End loop

## 3.3 Recolouring of images for validation purposes

Recolouring of images was also done using R version 3.6 and the *EBImage* package. This process uses the region-growing algorithm stated above. It tests the colour values of all the pixels in an image and reassigns the colour value of a pixel if the CIE ΔE2000 distance (relative to a tested seed colour) is below the defined threshold value. In this manner all the colours in an image are reassigned the value of their perceptually unique bin and a perceptually unique raster of the image is made. The following pseudocode was used to recolour images.

Declare double: *d*

Declare *L*a*b** array (double): *L_RG_*, *L_Image_*

Declare *L*a*b** vector (double): *L_q_*, *L_t_*

Declare integer *n_i_*, *n_r_*

1. Open *Region Grow data* and corresponding *Image data*
2. Covert *Region Grow data* hexadecimal code to *L*a*b** colour space (*L_RG_*).
3. Covert *Image data* hexadecimal code to *L*a*b** colour space (*L_Image_*).
4. Set *n_r_* = number of row values in *L_RG_ (Region grow data)*
5. Set *n_i_* = number of pixels in *L_Image_ (Image data)*
6. Loop: Repeat for less than or equal to *n_r_*

Loop: Repeat for less than or equal to *n_i_*

Set *d* = 0

Set *L_s =_ L_RG_* [*n_r_*] (seed)

Set *L_q =_ L_Image_* [*n_i_*] (query)

Calculate *d* using ΔE2000 equation between *L_s_* and *L_q_*

if *d* <= 2 then

Assign *L_s_* value to *L_Image_* [*n_i_*]

Remove *L_Image_ = L_Image_* [-*n_i_*] from pool of values to test

End loop

End loop

1. Raster image and save

## 3.4 Weighted CIE ΔE2000 distance metric

The weighted CIE ΔE2000 distance metric was developed and implemented in R version 3.6. This metric works by using the ‘perceptually unique colour (PUC) tables’ generated by the region-growing algorithm. A ‘transport matrix’ is constructed by undertaking a full-rank comparison of all of the PUC values contained between two PUC-tables (images). The CIE ΔE2000 distance function is used to calculate a distance metric between each respective colour comparison. A ‘weighed distance value’ is then generated by multiplying the ΔE2000 distance value by the difference in frequency (as a percentage) of the respective PUC comparison. The closest match to a PUC value is considered as the minimum weighed distance value contained in each row of the ‘transport matrix’. The overall similarity between two PUC profiles (all the values in a PUC-table) can then be considered as the sum of the minimum weighed distance values (closest matches). This is similar to Wasserstein metric but uses the CIE ΔE2000 formula to define distance and factors in the frequency/abundance difference between colour values. The following pseudocode was used to generate the CIE ΔE2000 weighted distance matrix.

Declare double: *d*, *Freq_q_*, Freq_t_*, Freq_Diff_*

Declare integer: *q, t, nr_q_, nr_t,_*

Declare *L*a*b** array (double): *L_Query_*, *L_Target_*

Declare *L*a*b** vector (double): *L_s_*, *L_t_*

Declare array (double)*: Query_array*, *Target_array*

Declare array (double): *Transport_array* [*n_q_* , *n_t_*]

Declare vector (double): *vector_min* [*n_q_*]

Declare double: *cumulative sum*

Declare array (double): *Distance_array* [*q* , *t*]

Nested Loop:

1. Loop: For *q* in number of *Region Grow data tables*

Loop: For *t* in number of *Region Grow data tables*

Set *Query_array* = *Region Grow data table*[*q*]

Set *Target_array* = Open *Region Grow data table*[*t*]

Set *nr_q_* = row dimension of *Query_array*

Set *nr_t_* = row dimension of *Target_array*

Set *L_Query_* = Covert *Query_array* hexadecimal codes to *L*a*b** colour space

Set *L_Target_* = Covert *Target_array* hexadecimal codes to *L*a*b** colour space

Nested Loop:

- - 1. Loop: Repeat for less than or equal to *nr_q_*
    2. Loop: Repeat for less than or equal to *nr_t,_*

Set *d* = 0

Set *L_q_ = L_Query_* [*nr_q_*]

Set *L_t_ = L_Target_* [*nr_t_*]

Calculate *d* using ΔE2000 equation between *L_q and_ L_t_*

Set *Freq_q_* = *Query_array*[*nr_q_*] (Query frequency data)

Set *Freq_t_* = *Target_array*[*nr_t_*] (Target frequency data)

Calculate *Freq_Diff_ =* *Freq_q_ - Freq_t_*

if *d*  = 0 then

Set *Transport_array* [*n_q_* , *n_t_*] =  *Freq_Diff_*

else

Set *Transport_array* [*n_q_* , *n_t_*] = *d** *Freq_Diff_*

End loop

End loop

Calculate *vector_min* = Find minimum value in each row of *Transport_array*

Calculate *cumulative sum* = sum of all values in *vector_min*

Set *Distance_array* [*q*, *t*] = *cumulative sum*

End loop

End loop

1. Save distance matrix *Distance_array*
